# Change Editorial

**Published:** 1871-12

**Authors:** 


### CHANGE EDITORIAL.

With the issue of the present number of the DENTAL REGISTER the editorial connec ion of Dr. WATT ceases. Nevertheless, we hope frequently to have contributions from his pen for its pages. This step has been rendered necessary by his removal from Cincinnati; and, in addition to this, his time is so fully occupied that he does not wish to be held responsible for regular contributions, and he desires to have as much freedom from care and responsibility as possible, that he may have the greater opportunity for recuperation.

The editorial control and management of the REGISTER will be entirely in charge of the former senior editor, to whom all m.tter and communications for its pages should be addressed.

